# Case of Abscess through the Lumbar Region of the Back—Discharge of Twenty Stones

**Published:** 1843-07-08

**Authors:** A. H. Perkins

**Affiliations:** Ayletts, King Wm. co, Va.


### CASE OF ABSCESS THROUGH THE LUMBAR REGION OF THE BACK—DISCHARGE OF TWENTY STONES.

BY A. H. PERKINS, M. D.

To the Editor of the Medical Examiner.

*Sir,*—I send you enclosed, two (of twenty) stones discharged by an abscess through the lumbar region of the back.

The patient is a man of apparently good constitution, and has enjoyed moderate health for many years. About ten years since, he says, he had frequent pains in his back, and much acidity of stomach, which were removed by treatment.

In February, 1842, I saw him, in company with Dr. D. Tabb, of Matthews county. A few months before a swelling had commenced in his back, just below the kidney of the left side, and continued to increase until this time. Poultices, &c., had been used, and now a fluid was perceptible to the touch. An abscess lancet was introduced to the depth of an inch through the swollen part, and a considerable quantity of serous blood escaped, and continued to escape for several days. The poultices were reapplied, but owing to irregularity of treatment, a sinus of an unhealthy character was formed which was cured with port wine injections, caustic, &c. No disease of the kidney was suspected. The digestion was good, urine tolerably clear, and the gentleman able to attend to his office of Sheriff, throughout the whole period, and it was thought advisable to close the wound. It continued, however, tender with occasional pain in the part, until December following, when it commenced rising again. Broke in March following and discharged the stones above mentioned. The pain in the back was intermittent throughout the whole period, not much increased by pressure; the constitutional symptoms very slight; and the urine not more sedimentous than occurs in slight febrile affections, and not constant in that situation.

The place is now open, discharging pus with a grisly substance protruding from it. I advised the application of caustic; constitutional treatment was refused, the patient being a Thompsonian.

*Ayletts, King Wm. co. Va.*, June 6, 1843.

